# Segregation of Odor Identity and Intensity during Odor Discrimination in *Drosophila* Mushroom Body

**DOI:** 10.1371/journal.pbio.0050264

**Published:** 2007-10-02

**Authors:** Shouzhen Xia, Tim Tully

**Affiliations:** Cold Spring Harbor Laboratory, Cold Spring Harbor, New York, United States of America; Austrian Academy of Sciences, Austria

## Abstract

Molecular and cellular studies have begun to unravel a neurobiological basis of olfactory processing, which appears conserved among vertebrate and invertebrate species. Studies have shown clearly that experience-dependent coding of odor identity occurs in “associative” olfactory centers (the piriform cortex in mammals and the mushroom body [MB] in insects). What remains unclear, however, is whether associative centers also mediate innate (spontaneous) odor discrimination and how ongoing experience modifies odor discrimination. Here we show in naïve flies that G_αq_-mediated signaling in MB modulates spontaneous discrimination of odor identity but not odor intensity (concentration). In contrast, experience-dependent modification (conditioning) of both odor identity and intensity occurs in MB exclusively via G_αs_-mediated signaling. Our data suggest that spontaneous responses to odor identity and odor intensity discrimination are segregated at the MB level, and neural activity from MB further modulates olfactory processing by experience-independent G_αq_-dependent encoding of odor identity and by experience-induced G_αs_-dependent encoding of odor intensity and identity.

## Author Summary

Considerable progress has been made in understanding how olfaction works as the receptor proteins, sensory neurons, and brain circuitry responsible have become increasingly well-characterized. However, olfactory processing in higher brain centers, where neuronal activity is assembled into the perception of odor quality, is poorly understood. Here, we have addressed how the mushroom body (MB)—a secondary olfactory center—is involved in olfactory discrimination. We manipulated the MB by ablation, disruption of synaptic transmission, and interruption of key cellular signaling molecules in naïve flies and in flies trained to discriminate odors. We first show that although both odor identity and intensity are encoded in the MB, only the former requires G_αq_-dependent signaling and is necessary for naïve flies to spontaneously discriminate different odors. We then show that training flies to alter their olfactory response requires G_αs_-mediated signaling in MB for both odor intensity and odor identity. We have thus identified (i) segregation of odor identity and odor intensity at the MB level in naïve flies and (ii) different G-protein-dependent signaling pathways for spontaneous versus experience-dependent olfactory discrimination.

## Abstract 3

Experience-dependent modification of odor identity and intensity occurs in the mushroom body (MB) of flies exclusively via G_αs_-mediated signaling. In contrast, G_αq_-mediated signaling in MB modulates spontaneous discrimination of odor identity but not odor intensity.

## Introduction

Naturally, most odors are experienced as complex mixtures in the environment. Consequently, an animal's ability to discriminate between two odor cues reflects the interplay between spontaneous and experience-dependent processes [1–3]. Odor discrimination traditionally has been assessed either by using conditioning procedures to reveal that an animal can learn a conditioned response for one odor over another and therefore can discriminate them (e.g., [1,4,5]) or by demonstrating experience-dependent olfactory adaptation (e.g., [6–9]). When trying to identify the underlying cellular and molecular mechanisms for odor discrimination and associative learning, however, this approach becomes confounding. The cAMP signaling pathway, for instance, has been shown to be involved in both olfactory processing and associative learning in mammals and in *Drosophila* [10–14]. Similarly, experience-dependent changes in odor discrimination have been shown to occur in the piriform cortex of vertebrates [1,5,15–18] and in the mushroom body (MB) of *Drosophila* [19–22], both of which are activated by odor stimulation in naïve animals [23–28].

Odors elicit a variety of behavioral responses in *Drosophila* via a relatively simple but sensitive olfactory system readily accessible to genetics [29]. A number of behavioral assays have been reported for screening single-gene mutations that cause defects in olfactory function and in olfactory associative learning. The predominant learning assay employs Pavlovian conditioned discrimination between two odors [30]. A behavioral mutant might display an abnormal behavioral response in this assay because of (i) a sensorimotor deficit to odor(s), (ii) a sensorimotor deficit to footshock, (iii) a sensorimotor deficit to odor discrimination, or (iv) a deficit in the association of odor and footshock. Traditionally, sensorimotor deficits to odors and to footshock have been assessed in a T-maze using an olfactory acuity assay ([31]; also see below) and a shock reactivity assay [32]. For the former, flies are given a choice between an odor and air. For the latter, flies are given a choice between footshock versus no footshock. To date, mutants are considered to be associative learning/memory mutants if they behave normally in these two sensorimotor assays. Significantly, no assay for sensorimotor deficits in odor discrimination per se has yet been developed.

Ultimately to address this issue, we have developed two novel assays to measure discrimination of odor intensity and odor identity with a T-maze [30] in naïve flies. Work on odor intensity discrimination in naïve flies then prompted us to develop a third novel assay to measure conditioned discrimination of odor intensity (cf. [33,34]). Importantly, genetic manipulations in *Drosophila* can be used to link these behavioral responses to specific molecular mechanisms. By disrupting MB function in four distinct ways (i.e., developmental lesion of MB, silencing of synaptic transmission from MB, RNA interference [RNAi]–mediated suppression of G_αq_-mediated signaling, and disruption of G_αs_-mediated signaling in MB) and assessing the effects with each of our four distinct behavioral assays, we have asked whether a secondary olfactory center (MB) is required for spontaneous odor discrimination and whether the same cAMP and IP_3_ signaling pathways that are known to mediate odor transduction in peripheral sensory neurons [10,11] also regulate olfactory processing in the central nervous system. Our results demonstrate that (i) MB is required for spontaneous discrimination of odor identity but not odor intensity via a G_αq_-dependent signaling pathway and (ii) MB is required for conditioned discrimination of odor identity or odor intensity via a G_αs_-dependent signaling pathway.

## Results

To explore how *Drosophila* MB participates in spontaneous versus conditioned odor discrimination, we designed three novel odor discrimination assays—a spontaneous odor intensity assay, a spontaneous odor identity assay, and a conditioned odor intensity assay (see Protocol S1 for details)—to use along with our original conditioned odor identity assay [30]. With these four behavioral protocols, we were able to quantify (i) discrimination of odor intensity in naïve flies (Figure S1A), (ii) discrimination of odor identity in naïve flies (Figures S1B, S2, and S3), (iii) discrimination of odor intensity in conditioned flies (Figure S1C), and (iv) discrimination of odor identity in conditioned flies (Figure S1D).

We then disrupted MB structure or function in four distinct ways and assessed their effects with each of these four behavioral assays. First, we lesioned MB using chemical ablation [20]. When hydroxyurea feeding is restricted to the first few hours after hatching, this method kills proliferating cells during development and results in a dramatic reduction of adult MB and loss of a portion of the antennal lobes (ALs; Figure S4). Second, we acutely blocked dynamin-dependent synaptic transmission from MB of transgenic flies by using three different PGAL4 enhancer-trap drivers to express UAS-*shi^ts1^* (shi^ts^) preferentially in MB [35]. Third, we disrupted expression of *G_αq_* in MB of transgenic flies by using the same PGAL4 drivers to express a UAS-ds*G_αq_* (RNAi) construct [36]. We then duplicated the essential experiment with a second RNAi (UAS-*dG_q_* ^1F1^) transgene [37]. Both UAS-ds*G_αq_* and UAS-*dG_q_* ^1F1^ produced severe knockdowns of G_αq_ expression (Figure S5; see also [36,37]). Fourth, we disrupted G_αs_ signaling in MB of transgenic flies by using the same PGAL4 drivers to express UAS-*G_αs_* ^Q215L^ (G_αs_*), a constitutively active stimulatory subunit of guanosine triphosphate-binding protein [19]. As a genetic control, wild-type *G_αs_* transgene (UAS-*G_αs_* ^+^) was overexpressed using the same PGAL4 drivers.

### Spontaneous Odor Intensity Discrimination Does Not Require the Normal Function of MB

**Figure 1:**
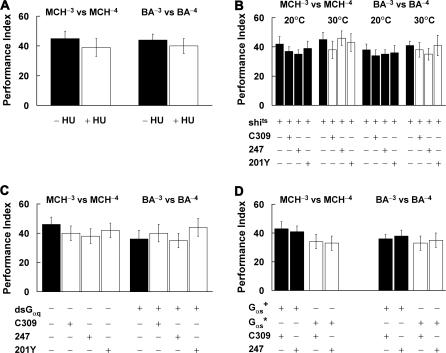
Spontaneous Odor Intensity Discrimination Does Not Require the Normal Function of MB In this and the following figures, all control groups are shown in black bars, and the experimental groups in white bars, or blue when statistically significantly different from the control (*p* < 0.05), and all experiments were done in a balanced and experimenter-blind manner. Spontaneous intensity discrimination between different concentrations of MCH (left panels) or BA (right panels) was not affected (A) by MB ablation (+HU), (B) by transient block (30 °C) of temperature-sensitive (20 °C, permissive; 30 °C, restrictive) *shibire*-dependent synaptic transmission using three different MB PGAL4 drivers (C309, 247, and 201Y), (C) by RNAi-mediated disruption of *G_αq_* using the same MB PGAL4 drivers, or (D) by overexpression of constitutively active G_αs_* (with *G_αs_* ^+^ as controls) using two MB PGAL4 drivers (C309 and 247). *n =* 4 PIs for all groups.

MB ablation had no effect on flies' spontaneous odor intensity discrimination (Figure 1A) and avoidance of individual odors (Table S1). To check whether neuronal activity from MB is acutely involved with the spontaneous odor response, synaptic transmission from MB was transiently silenced by driving transgenic expression of shi^ts^ with several MB-specific PGAL4 drivers. C309 and 247 drive transgenic expression in all lobes of MB, while 201Y drives transgenic expression preferentially in γ-lobes ([19,38]; see also Figure S4). Consistent with MB ablation, transient silencing of synaptic transmission from MB left spontaneous odor intensity discrimination (Figure 1B) and avoidance of individual odors (Table S1) unchanged. Similarly, neither disruption of *G_αq_* by overexpression of UAS-ds*G_αq_* nor disruption of *G_αs_* by overexpression of G_αs_* with the same PGAL4 drivers interfered with flies' spontaneous odor intensity discrimination (Figure 1C and 1D) or avoidance of individual odors (Table S1). These observations indicate that MB is not required for discrimination of odor intensity or perception of individual odors in naïve flies. Such olfactory processing perhaps is accomplished in ALs or other brain regions, such as the lateral horn [39].

### Spontaneous Odor Identity Discrimination Requires Synaptic Transmission from MB and Depends on G_αq_ Signaling

**Figure 2:**
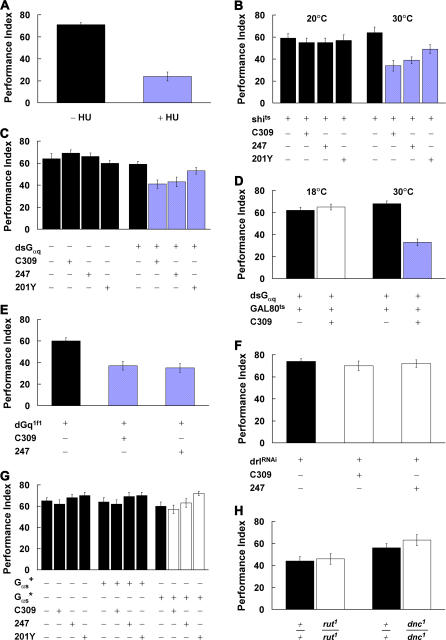
Spontaneous Odor Identity Discrimination Requires Synaptic Transmission from MB and Depends on G_αq_ Signaling (A−E) Spontaneous odor identity discrimination between MCH and BA was severely disrupted (A) by MB ablation (+HU), (B) by transient block (30 °C) of synaptic transmission from MB in C309/shi^ts^, 247/shi^ts^, or 201Y/shi^ts^ flies, (C) by developmental disruption of *G_αq_* in MB of ds*G_αq_*/C309, ds*G_αq_*/247, and ds*G_αq_*/201Y flies, (D) by adult-specific, physiological disruption of *G_αq_* in MB of ds*G_αq_*, GAL80^ts^/C309 flies at restrictive (30 °C for 3 d) but not permissive (18 °C for 3 d) temperature, or (E) by developmental disruption of *G_αq_* in MB of *dG_q_* ^1F1^/C309 or *dG_q_* ^1F1^/247 flies. (F−H) Spontaneous odor identity discrimination was not affected (F) when the *drl* gene was knocked down in MB via RNAi-mediated disruption (T. T., H. Tang, and M. Regulski, unpublished data) in *drl* ^RNAi^/C309 or *drl* ^RNAi^/247 flies, (G) when *G_αs_* was disrupted in MB of G_αs_*/C309, G_αs_*/247, or G_αs_*/201Y flies, or (H) in *rut* and *dnc* mutants. *n =* 4 PIs for all groups.

MB ablation (Figure 2A) and transient silencing of synaptic transmission from MB (Figure 2B) disrupted flies' spontaneous odor identity discrimination, suggesting that MB is acutely involved with the process. Some spontaneous response remained, in particular after MB ablation, suggesting other brain regions may also be involved in the discrimination of odor identity. This is consistent with the fact that projection neurons do not form synaptic collaterals with the calyx of MB after hydroxyurea-based ablation, whereas their arborizations in the lateral horn remain [40].

RNAi-mediated disruption of *G_αq_* in MB with the same PGAL4 drivers was sufficient to interfere with spontaneous odor identity discrimination (Figure 2C). To rule out the possibility that a developmental defect underlay this adult defect in the spontaneous odor identity response, the *tub*-GAL80^ts^ transgene was used along with the binary PGAL4/UAS system [21]. Groups of flies, raised at 18 °C, were kept for 3 d either at 18 °C (permissive for inhibition of PGAL4 by GAL80^ts^) or 30 °C (restrictive for GAL80^ts^ function) before testing of spontaneous odor identity discrimination. The performance was significantly disrupted in transgenic C309/+; ds*G_αq_*, GAL80^ts^/+ flies at the restrictive but not at the permissive temperature (Figure 2D), suggesting an adult-specific physiological role for *G_αq_* during spontaneous odor identity discrimination. To rule out the possibility of an off-target RNAi effect (cf. [41,42]), a second RNAi transgene construct, UAS-*dG_q_* ^1F1^ [37], was evaluated. Again, we saw significant disruption of spontaneous odor identity discrimination (Figure 2E). As a further control for a more general “poisoning” of neuronal function by RNAi, we showed that expression of a UAS-*drl* ^RNAi^ transgene in MB had no effect on this odor response (Figure 2F). Collectively, these results demonstrate, to our knowledge for the first time, an adult-specific role in the central nervous system for G_αq_ signaling during discrimination of odor identity in naïve flies.

In contrast to the effect of *G_αq_,* disruption of G_αs_ signaling in MB had no effect on spontaneous odor identity discrimination (Figure 2G). Consistent with this observation, the *rutabaga* and *dunce* mutants, in which two other components of the cAMP signaling pathway (i.e., adenylyl cyclase and cAMP-specific phosphodiesterase, respectively) are defective [43,44], showed normal spontaneous odor identity discrimination (Figure 2H). Taken together, these observations suggest that (i) odor identity discrimination in naïve flies occurs independently of the cAMP signaling pathway and (ii) G_αq_-dependent signaling in MB specifically contributes to this response.

### Conditioned Odor Intensity Discrimination Requires Synaptic Transmission from MB and Depends on G_αs_ Signaling

**Figure 3:**
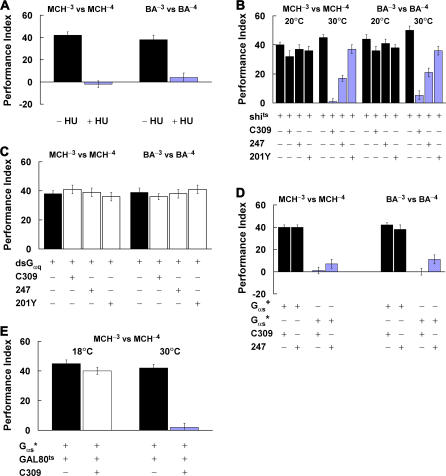
Conditioned Odor Intensity Discrimination Requires Synaptic Transmission from MB and Depends on G_αs_ Signaling Conditioned odor intensity discrimination between different concentrations of MCH (left panels) or BA (right panels) was (A) abolished by MB ablation (+HU), (B) disrupted by transient block (30 °C) of synaptic transmission from MB in C309/shi^ts^, 247/shi^ts^, or 201Y/shi^ts^ flies, (C) not affected by disruption of *G_αq_* in MB of ds*G_αq_*/C309, ds*G_αq_*/247, or ds*G_αq_*/201Y flies, (D) severely affected by developmental disruption of *G_αs_* in MB of G_αs_*/C309 or G_αs_*/247 flies, and (E) abolished in an adult-specific manner by disruption of *G_αs_* (at restrictive temperature, 30 °C, for 3 d) in MB of G_αs_*, GAL80^ts^/C309 flies. *n =* 6 PIs for each group.

MB ablation completely abolished conditioned odor intensity discrimination (Figure 3A), suggesting that conditioned odor intensity discrimination requires an intact MB. Similarly, transient block of synaptic transmission from MB severely disrupted conditioned odor intensity discrimination (Figure 3B), suggesting an acute role for neuronal activity in MB for this conditioned response. Finally, disruption of G_αs_ but not G_αq_ signaling also severely disrupted conditioned odor intensity discrimination, and the effect was adult-specific and physiological (Figure 3C–3E). Considering that spontaneous odor intensity discrimination occurs independent of MB (Figure 1), these observations reveal that (i) there is an “anatomical dissection” for odor intensity discrimination between naïve and conditioned flies and (ii) G_αs_-mediated neural plasticity in MB modulates odor intensity discrimination in conditioned but not in naïve flies.

### Conditioned Odor Identity Discrimination Requires Synaptic Transmission from MB and Depends on G_αs_ Signaling

**Figure 4:**
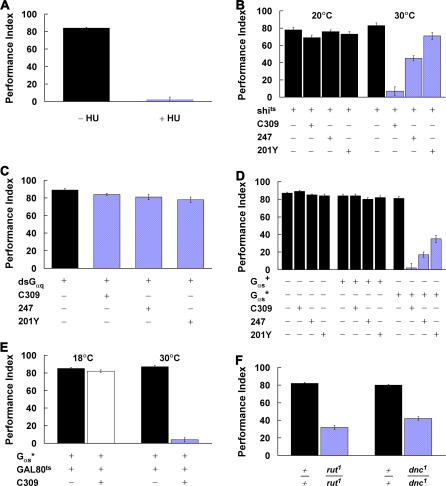
Conditioned Odor Identity Discrimination Requires Synaptic Transmission from MB and Depends on G_αs_ Signaling Conditioned odor identity discrimination between MCH and BA was (A) abolished by MB ablation (+HU), (B) disrupted by transient block (30 °C) of synaptic transmission from MB in C309/shi^ts^, 247/shi^ts^, or 201Y/shi^ts^ flies, (C) mildly affected by disruption of *G_αq_* in MB of ds*G_αq_*/C309, ds*G_αq_*/247, or ds*G_αq_*/201Y flies, (D) severely diminished by developmental disruption of *G_αs_* in MB of G_αs_*/C309, G_αs_*/247, or G_αs_*/201Y flies, (E) abolished by adult-specific, physiological disruption of *G_αs_* in MB of G_αs_*, GAL80^ts^/C309 flies at restrictive (30 °C) but not permissive (18 °C) temperature, and (F) disrupted in *rut* or *dnc* mutants. *n =* 6 PIs for each group.

MB ablation abolished conditioned odor identity discrimination (Figure 4A), confirming a previous report [20]. Conditioned odor identity discrimination with a different odor pair, 3-octanol and 4-methyl-cyclohexanol (MCH), has been reported to be abolished by transient silencing of synaptic transmission from MB in C309/shi^ts^ flies [22] and mildly disrupted even at the permissive temperature in 247/shi^ts^ flies [21]. Here, we confirmed and extended such observations by showing that conditioned odor identity discrimination between MCH and benzaldehyde (BA) was severely disrupted by transient silencing of synaptic transmission from MB in C309/shi^ts^ and 247/shi^ts^ flies and mildly disrupted in 201Y/shi^ts^ flies at restrictive temperature (Figure 4B).

Disruption of *G_αq_* in MB had only a very mild effect on conditioned odor identity discrimination (Figure 4C). Considering that the same flies were defective for spontaneous odor identity discrimination, this mild effect may be indirect.

Conditioned odor identity discrimination between 3-octanol and MCH has been reported to be disrupted when G_αs_* but not *G_αs_* ^+^ is overexpressed in MB [19]. Here we again confirmed our previous finding with 3-octanol and MCH (data not shown) and extended it by showing a similar effect with a different odor pair, MCH and BA (Figure 4D). Again, to rule out the possibility that a developmental defect underlay this effect on conditioned odor identity discrimination, the *tub*-GAL80^ts^ transgene was used to restrict expression of G_αs_* in adults. Conditioned odor identity discrimination was abolished in transgenic C309/+; G_αs_*, GAL80^ts^/+ flies when kept at the restrictive but not at the permissive temperature (Figure 4E), suggesting an adult-specific physiological role for G_αs_ during conditioned odor identity discrimination. Consistent with this notion, *rutabaga* and *dunce* mutants showed disrupted conditioned odor identity discrimination (Figure 4F), as demonstrated long ago [30]. These results are strikingly different from those for spontaneous odor identity discrimination, revealing a “genetic dissection” for odor identity discrimination between naïve and conditioned flies. In MB, G_αq_ signaling mediates odor identity discrimination in naïve flies, while G_αs_ signaling mediates odor identity discrimination in conditioned flies.

## Discussion

The use of a Pavlovian conditioned odor discrimination assay [30] over the past 20 y has helped to establish the view that G-protein-mediated cAMP signaling in MB subserves this form of associative learning [45]. Now, by developing additional behavioral measures, we are able to dissect odor discrimination into four functionally distinct components: the spontaneous response to odor intensity, the spontaneous response to odor identity, the conditioned response to odor intensity, and the conditioned response to odor identity. Our results refine our view of olfactory behavior as it relates to MB function, revealing an anatomical and molecular (genetic) dissection between odor identity and odor intensity in this higher brain center (Table 1). We have established MB as one important anatomical site for odor identity discrimination in naïve flies (Figure 2). Odor intensity discrimination, in contrast, does not require MB at all in naïve flies (Figure 1). Within MB, different G-protein-mediated signaling pathways distinguish odor identity discrimination between naïve and conditioned flies. G_αq_-dependent signaling regulates spontaneous responses to odor identity, while G_αs_-dependent signaling is involved in conditioned responses to odor identity (Figures 2 and 4). Strikingly, disruption of G_αs_-dependent signaling produces similar effects on conditioned responses to both odor identity and odor intensity, while disruption of G_αq_ has little or no effect on these behavioral responses (Figures 3 and 4).

**Table 1:**
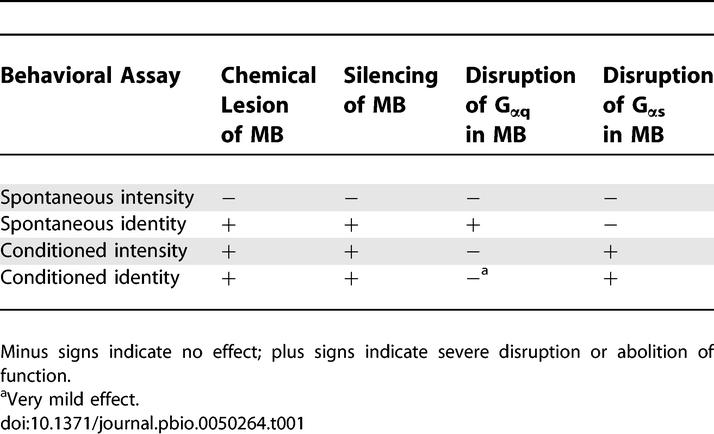
Effects of Four Experimental Manipulations of MB Function on Four Behavioral Assays of Odor Discrimination

### Segregation of Odor Identity and Odor Intensity in Naïve Flies

Odor discrimination is usually assayed in the context of olfactory adaptation (perceptual learning) or conditioning (associative learning) because of the difficulty in directly evaluating the process in naïve animals (e.g., [1,4–9]). Our spontaneous odor identity discrimination assay, where naïve flies are allowed to recognize and discriminate a second odor from a saturated odor as the background (Figures S1–S3), is analogous to a saturation discrimination paradigm in *Caenorhabditis elegans,* the only other known discrimination assay developed for naïve animals [46]. When an odor is saturated in intensity, higher concentrations produce no further change in response (Figure S2A and S2C). Against a saturated background odor, a “foreground” odor may be represented in two possible ways. First, the odor may activate additional receptors in the sensory neurons, leading to activation of additional subsets of glomeruli and the corresponding MB neurons (cf. [47]). Second, the foreground odor may activate additional neural processes or signaling pathways, leading to activation of additional subsets of MB neurons. Either way, flies clearly recognize the presence of the foreground odor and discriminate it from a background odor (Figure S2C). More importantly, our saturation assay appears to quantify odor identity discrimination independent of odor intensity discrimination in naïve flies (Figure S2C).

MB cells respond differently to different odors, and some of them are reported to be concentration-invariant [23,28], but their behavioral function in odor identity and intensity discrimination is unknown. It also remains unclear whether the piriform cortex encodes odor identity in vertebrates [5,15,16]. Hydroxyurea-induced ablation or shi^ts^-dependent silencing of MB severely diminishes (but does not abolish) spontaneous odor identity discrimination but not odor intensity discrimination (Figures 1 and 2; Table 1), suggesting that MB specifically contributes to behavioral responses to odor identity in naïve flies. The effect for our particular odor pair is partial, suggesting that processing of odor identity (but not intensity) may be of major importance for naïve flies, and thus needs to recruit more than one perceptual center (i.e., MB). This notion leads to the conclusion that odor identity and odor intensity are distinct and must be processed, at least in part, through different neuronal circuitries in the central brain. Our demonstration that MB mediates spontaneous responses to odor identity is particularly important for understanding olfactory processing in central olfactory centers, where neuronal activity may be recruited to generate the perception of odor identity (cf. [48]).

Nonetheless, increases in odor concentration recruit additional glomeruli at ALs and lead to changes in the firing patterns of projection neurons, similar to those caused by changes in odor identity [28,49]. Consequently, the spatiotemporal codes for odor identity and intensity may be confounded in ALs. Such observations highlight our hypothesis that MB serves to segregate representations of odor identity and odor intensity. Such an anatomical segregation between odor identity and intensity in a higher brain center like MB is consistent with the observations that (i) odor identity and intensity assays engage different (although overlapping) central brain regions in human [16] and (ii) odor identity and structure are coded separately within the piriform cortex [15]. Therefore, olfactory information in *Drosophila* may be decomposed into various features and processed in distinct central perceptual centers (also see [50]), as in other sensory systems [51–53].

### Segregation of Odor Identity and Odor Intensity in Naïve versus Conditioned Flies

Little is known about G_αs_- or G_αq_-mediated signaling pathways in secondary olfactory centers in spite of the facts that (i) G_αq_ appears to be involved in olfactory signal transduction in sensory neurons [36], (ii) G_αs_ is involved in conditioned odor identity discrimination in *Drosophila* [19], and (iii) these higher centers clearly are involved in experience-dependent modification of odor discrimination in vertebrates [1,5,15–18] and in *Drosophila* [19–22]. The fact that MB is required (i) for spontaneous responses to odor identity but not intensity (Table 1) and (ii) for conditioned responses to odor identity [19–22] prompted us to check G_αs_- versus G_αq_-mediated signaling pathways in MB.

Disruption of *G_αq_* in MB affects the spontaneous response to odor identity but unexpectedly affects the conditioned response to odor identity only minimally. Disruption of *G_αs_* in MB, on the other hand, affects the conditioned response to odor identity but does not affect the spontaneous response to odor identity (Table 1). Such (i) exclusion of G_αq_ from the conditioned response to odor identity and of G_αs_ from the spontaneous response to odor identity and (ii) specificity of G_αq_ for the spontaneous response to odor identity and of G_αs_ for the conditioned response to odor identity constitute a dual dissection of spontaneous and conditioned odor identity discrimination. Moreover, the conditioned response to odor intensity is abolished by disruption of *G_αs_* in MB (Figure 3D), while the spontaneous response to odor intensity occurs independent of MB, suggesting different anatomical contributions to spontaneous and conditioned odor intensity discrimination. Such an anatomical/molecular dissection of spontaneous and conditioned odor responses suggests that the learned response occurs independently of the spontaneous response, questioning the traditional experiments where odor discrimination per se is evaluated in the context of olfactory learning.

Finally, the correspondence between the conditioned responses to odor intensity and odor identity when disrupted by MB ablation, MB silencing, or jamming of *G_αs_* is simply striking (Table 1). This general observation argues that, in contrast to the fact that the spontaneous responses to odor intensity or odor identity are segregated at the MB level, G_αs_-mediated neuronal plasticity in MB nonetheless underlies behavioral changes to both.

Our data collectively demonstrate that (i) odor identity encoded in MB contributes to olfactory discrimination through a G_αq_-dependent signaling process, whereas odor intensity encoded in MB exists but is not necessary for behavioral responses in naïve flies and (ii) G_αs_-mediated signaling in MB is exclusively involved in neural plasticity, which then modulates behavioral responses to both odor intensity and identity. Such segregation of odor identity and odor intensity at the MB level in naïve flies and identification of different G-protein-dependent signaling pathways for spontaneous and conditioned odor discrimination, combined with function imaging [23] and the recently established spatial map of olfactory representations in MB [47], make it possible to check how neuronal activity in MB is assembled into the perception of odor identity (cf. [48]).

## Materials and Methods

### Fly stocks.

Fly stocks included wild-type Canton-S *w^1118^* (CS10); the PGAL4 insertions 247 [38], C309, and 201Y; and UAS constructs UAS-*G_αs_* ^+^, G_αs_* [19], shi^ts^ [35], UAS-ds*G_αq_*-RNAi [36], and UAS-*dG_q_* ^1F1^ [37]. UAS-*GFP* ^S65T^ was obtained from the Bloomington *Drosophila* Stock Center (http://flystocks.bio.indiana.edu/). All stocks were “Cantonized” by outcrossing the heterozygous virgins to *w^1118^* (CS10) males for six generations, selecting for the mini-white^+^ eye color associated with each P element transposant.

### Odorants.

The following odors were used: two alcohols, 3-octanol and MCH; one aromatic compound, BA; one ester, ethyl acetate; one ketone, diacetyl; one amine, triethylamine; and thiazole. Some chemically similar odors were also tested including (R)-(−)-carvone and (S)-(+)-carvone, (R)-(−)-octanol and (S)-(+)-octanol, pentanol, 2-hexanol, and hexanol. All odors were dissolved in heavy mineral oil (Fisher Scientific, https://www.fishersci.com/) and delivered to the training chamber or the two T-maze arms with a “test bubbler” [54].

For conditioned discrimination, the concentration of MCH was chosen as 1.0 × 10^−3^ (in mineral oil [v/v]) [54] or alternatively 1.0 × 10^−4^. Then the relative concentrations of BA (0.4 × 10^−3^ or 0.4 × 10^−4^) were determined so that naïve flies distributed themselves 50:50 in the T-maze when given a choice between MCH and BA. For odor avoidance, MCH and BA were tested at concentrations from 10^−6^ to 10^−1^. For saturation discrimination, the concentrations were much higher and varied with odors as shown in Table S2.

### Behavioral assays.

To assess the flies' ability to sense individual odors, “fresh” air was delivered in one T-maze arm and odor (MCH or BA at different concentrations; Table S1) was delivered in the other, with all other parameters identical to those used during the traditional Pavlovian conditioned discrimination assay ([30]; and see below). Groups of about 100 naïve flies were lowered to the center of the T-maze, and their odor avoidance was quantified [31].

To assay spontaneous intensity discrimination, groups of about 100 naïve flies were given a choice in the T-maze between higher (i.e., 10^−3^) and lower (i.e., 10^−4^) concentrations of the same odor (either MCH or BA). Two groups of flies were always tested in one complete run to produce a pure measure of spontaneous intensity discrimination for naïve flies (Figure S1A). The first group was given the higher concentration in the left T-maze arm. A second, reciprocal group of naïve flies then was given the higher odor concentration in the right T-maze arm.

To assay spontaneous identity discrimination, groups of naïve flies were given a choice between a saturated background odor and a mixture containing the same background and a second odor. Crucially, the second odor was always weaker than or equivalent to the saturated background in intensity, and the background odor was “saturated” for intensity, thereby eliminating intensity differences from this assay (see Protocol S1 for details). Again, two groups of flies were always tested in one complete run to produce a pure measure of spontaneous identity discrimination for naïve flies (Figure S1B). The first group of naïve flies was exposed to a saturating concentration of the background odor (MCH^S^) plus an “equivalent” concentration of a second odor (BA^E^) in the left T-maze arm and the background (MCH^S^) only in the right T-maze arm. For the reciprocal group of naïve flies, the saturated background (MCH^S^) alone was presented in the left T-maze arm, and the mixture of the background and the second odor (MCH^S^ + BA^E^) presented in the right arm.

To assay conditioned intensity discrimination, groups of flies were first subjected to associative conditioning with different concentrations of the same odors before being tested for their acquired response to the different intensities. Again, two groups of flies were always tested in one complete run to produce a pure measure of acquired intensity discrimination for conditioned flies (Figure S1C). The first group of about 100 flies was first exposed to the higher odor concentration (10^−3^ of either MCH or BA; CS+) and subjected to footshock (US) for 60 s and then (after 45 s of air alone) exposed to the lower concentration (10^−4^ of either BA or MCH; CS−) without foot shock for another 60 s. A second reciprocal group of flies was trained with the lower odor concentration as the CS+ and the higher odor concentration as the CS−.

We also assayed conditioned identity discrimination, which corresponds to the traditional conditioned discrimination assay of Tully and Quinn [30] with minor modifications [54]. Again, two groups of flies were always tested in one complete run to produce a pure measure of acquired identity discrimination for conditioned flies (Figure S1D). The first group of about 100 flies was exposed sequentially to the CS+ (MCH) paired with footshock (US) and to the CS− (BA) without footshock. A second reciprocal group of flies was trained with BA as the CS+ and MCH as the CS−.

### Statistics.

Due to the nature of their mathematical derivation, performance indices (PIs) are distributed normally. Hence, the data were evaluated via one- or two-way ANOVAs. Subsequent pairwise planned comparisons were adjusted for experiment-wise error (α′), keeping the overall α equal to 0.05. All data are presented as mean ± standard error of the mean.

### Disruptions of MB structure or function.

MB ablation was achieved by feeding newly hatched larvae with hydroxyurea for about 6 h [20]. As a control, newly hatched larvae were fed with yeast suspension for 6 h.

Silencing of MB was achieved with the shi^ts^ transgene, which reversibly interferes with neuronal transmission in a temperature-dependent, dominant-negative fashion when overexpressed in a wild-type background [35]. shi^ts^ was crossed with C309 (C309/shi^ts^), 247 (247/shi^ts^), and 201Y (201Y/shi^ts^) males and grown at 18 °C throughout development. As a genetic control, shi^ts^ transgenic flies also were crossed to wild-type flies (+/shi^ts^). For the spontaneous intensity and identity assays, naïve flies were tested at 20 °C (permissive) as controls, or shifted to 30 °C (restrictive) for 30 min to block neural activity from MB before testing. For the conditioned intensity and identity assays, the flies were trained and tested immediately after training at 20 °C as controls, or shifted to 30 °C for 30 min to acutely block the synaptic transmission from MB before training and testing.

Disruption of *G_αq_* was achieved by blocking the transcription of *G_αq_* in MB by combining a UAS-ds*G_αq_*-RNAi construct with the above MB-PGAL4 drivers in transgenic flies (ds*G_αq_*/C309, ds*G_αq_*/247, and ds*G_αq_*/201Y). For genetic controls, ds*G_αq_*/+, +/C309, +/247, and +/201Y flies were generated. As a confirmation, transcription of *G_αq_* was also blocked in MB by combining an independent RNAi transgene, UAS-*dG_q_* ^1F1^, with C309 and 247 in transgenic flies (*dG_q_* ^1F1^/C309 and *dG_q_* ^1F1^/247).

To disrupt *G_αs_,* a constitutively activated stimulatory heterotrimeric guanosine triphosphate-binding protein, G_αs_*, was overexpressed to “jam” G_αs_ signaling in MB by crossing G_αs_* flies with the above PGAL4 drivers (G_αs_*/C309, G_αs_*/247, and G_αs_*/201Y). As genetic controls, a wild-type *G_αs_* transgene was overexpressed using the same PGAL4 drivers (*G_αs_* ^+^/C309, *G_αs_* ^+^/247, and *G_αs_* ^+^/201Y). Further control genotypes included G_αs_*/+, *G_αs_* ^+^/+, +/C309, +/247, and +/201Y.

### Measure of the vapor concentration.

The total molecules of each odor or odor mixture were measured in a paired manner for the four different behavioral tests of Figure S2C with a PID detector (miniPID-2, Aurora Scientific, http://www.aurorascientific.com/). The measurement was repeated six times and averaged to produce the actual vapor concentration for each odor or odor mixture.

### Western blotting.

For Western blotting, the adult head was homogenized with lysis buffer and centrifuged at 14,000 rpm at 4 °C for 50 min, and then the supernatant was saved. Lysate proteins were electrophoresed on a 12% SDS-PAGE, then electroblotted onto PVDF membranes. Immobilized proteins were probed with rabbit polyclonal anti-Gq (1:500 dilution) antiserum (SC-392; Santa Cruz Biotechnology, http://www.scbt.com/), or rabbit polyclonal anti-actin (1:5,000 dilution) antibody (A5060; Sigma-Aldrich, http://www.sigmaaldrich.com/) as the loading control, and the membrane was incubated with HRP-conjugated goat anti-rabbit IgG secondary antibody (1:5,000 dilution). The positive signal was visualized with the ECL System (GE Healthcare, http://www.gehealthcare.com/). Quantification of Western blot results was performed by digital image analysis using an Epson (http://www.epson.com/) scanner and ImageJ (National Institute of Mental Health, http://www.nimh.nih.gov/); experiments were repeated six times for +/Elav, UAS-ds*G_αq_*/Elav, and UAS-*dG_q_* ^1F1^/Elav flies. The level of G_αq_ was normalized to the actin control.

### Whole-mount GFP expression.

The protocol for whole-mount GFP expression was described before [55]. Briefly, homozygous PGAL4 females were crossed to homozygous UAS-*GFP* ^S65T^ males. Three- to five-day-old heterozygous female progeny were examined for GFP expression patterns. The whole brains were carefully transferred to 4% paraformaldehyde for 30 min, then to 4% paraformaldehyde + 0.25% Triton X-100 (Fisher Scientific) for 30 min under mild vacuum. Brains were then soaked in FocusClear (CelExplorer Labs, http://www.celexplorer.com/) solution for 5 min and mounted in a drop of the same solution [55]. The whole-mount brains were imaged with a Zeiss (http://www.zeiss.com/) LSM 510 confocal microscope, and stacks of confocal images were taken through the full thickness of the central brain. The distance between successive images (*z*-axis distance) was adjusted for the refractive index mismatch of the air and mounting medium as described previously [55]. In some cases, frontal and dorsal projections were rendered with Amira 3.1 (Mercury Computer Systems, http://www.tgs.com/) after removing optical slices between the brain surface and MBs to better reveal internal structures.

## Supporting Information

Figure S1 The Behavioral Protocols for Different Odor Discrimination Assays To control for potential side bias in the T-maze, two reciprocal groups always were tested as one complete experiment. A PI for the complete experiment was defined as the average of the PIs (always calculated as the number of flies avoiding the measured variable [i.e., the high concentration for spontaneous odor intensity discrimination, the mixture of the saturated background and the foreground odor for spontaneous odor identity discrimination, or the CS+ concentration or odor for conditioned odor intensity and odor identity discrimination assays] minus that avoiding the other “control” variable [i.e., the low concentration, the saturated background alone, or the CS− concentration or odor in the relevant assays], divided by the total number of flies and finally multiplied by 100) from the two reciprocal groups.
(A) The spontaneous odor intensity discrimination assay for naïve flies. Naïve flies were allowed to choose between two different concentrations (C1 and C2) of the same odors (10^−3^ and 10^−4^ for MCH, or 0.4 × 10^−3^ and 0.4 × 10^−4^ for BA), with the higher concentration delivered to the left arm in one group and to the right arm in the reciprocal group.
(B) The spontaneous odor identity discrimination assay for naïve flies. Naïve flies were allowed to make a choice between a saturated background odor (e.g., MCH) and a mixture consisting of the same saturated odor and a second foreground odor (e.g., BA). The mixture was delivered to the left arm in one group and to the right arm in the reciprocal group to produce a pure measure of spontaneous odor identity discrimination.
(C) The conditioned odor intensity discrimination assay for trained flies. The flies were conditioned to avoid one of the two concentrations (C1 or C2) of the same odors (10^−3^ and 10^−4^ for MCH, or 0.4 × 10^−3^ and 0.4 × 10^−4^ for BA), with US (marked with red electric volt symbol) associated with the higher concentration in one group and the lower concentration in the reciprocal group. The higher concentration was always delivered to the left arm during testing to cancel out both spontaneous odor intensity response and side bias.
(D) The conditioned odor identity discrimination assay for trained flies. The flies were conditioned to avoid one of the two equivalent odors (i.e., both spontaneous identity and intensity responses are close to zero; 10^−4^ for MCH and 0.4 × 10^−4^ for BA), with US associated with MCH in one group and with BA in the reciprocal group. MCH was always delivered to the left arm for both reciprocal groups.
(681 KB TIF)

Figure S2 The Saturation Discrimination Assay for Measuring Spontaneous Odor Identity Discrimination in Naïve Flies (A) Saturation of the background odor. To saturate MCH as the background odor, naïve flies were allowed to choose in the T-maze between 2 × [MCH] versus 1 × [MCH]. As the concentration of MCH was increased, a threshold (i.e., about 10%) was reached at which flies would fail to recognize the intensity difference, thereby yielding a PI of zero. At that concentration, MCH was considered saturated for intensity.
(B) Determination of the equivalent intensity of the foreground odor. To determine the concentration of BA equivalent to that of the saturated MCH (MCH^S^; i.e., 15% throughout the study), naïve flies were given a choice between [BA] and MCH^S^. As the concentration of BA was increased, the distributions of flies in the T-maze approached 50:50, yielding a PI of zero. That concentration of BA, BA^E^ (about 1.5%), was considered equivalent to saturated MCH.
(C) The saturation discrimination assay was produced by presenting either MCH^S^ + BA^E^ versus MCH^S^ (black bar, chosen as our standard assay throughout the study) or MCH^S^ + BA^E^ versus 2 × MCH^S^ (grey bar) in the T-maze. Because MCH^S^ (15%) was saturated, flies produced a score close to zero when presented with 2 × MCH^S^ versus MCH^S^ (*p =* 0.18). Similarly, because BA^E^ (1.5%) was equivalent to MCH^S^, flies also produced a score close to zero when presented with BA^E^ versus MCH^S^ (*p =* 0.46). The non-zero scores (*p* < 0.0001) for the two discrimination groups indicate that naïve flies are able to recognize the presence of BA and discriminate it from the saturated background of MCH. Scores from these two discrimination assays were not different from each other (*p =* 0.79), further confirming that MCH was saturated for intensity. The actual vapor concentrations of the individual odors or the mixture of MCH^S^ + BA were quantified and are shown in the lower panel.
(D) Saturation discrimination is concentration-dependent. Naïve flies were allowed to discriminate [BA] from the MCH^S^ (15%) across a wide range of concentrations (0.4 × 10^−4^, 0.4 × 10^−3^, 0.4 × 10^−2^, 0.75 × 10^−2^, and 0.15 × 10^−1^). Odor discrimination improved as [BA] increased.
(E) Saturation discrimination is influenced by exposure time to odors. Naïve flies were given a discrimination assay testing MCH^S^ + BA^E^ versus MCH^S^ for different lengths of time (30, 45, 60, 90, and 120 s). Optimal scores were produced quickly (i.e., with 30 s). When the time was longer than 60 s, discrimination scores dropped substantially.
*n =* 4 PIs for each group.
(572 KB TIF)

Figure S3 Spontaneous Discrimination between Chemically Different Odors (A) Naïve flies were tested for their ability to discriminate 3-octanol, BA, ethyl acetate, diacetyl, triethylamine, and thiazole from MCH^S^ (15%; grey columns); or MCH, 3-octanol, ethyl acetate, diacetyl, thiethylamine, and thiazole from BA^S^ (10%; black columns) as the saturated background odor (see Table S1 for concentrations of all odors).
(B) Reciprocally, naïve flies were tested for their ability to discriminate MCH^E^ (grey columns) and BA^E^ (black columns) from saturated 3-octanol, ethyl acetate, diacetyl, and thiazole as the background.
*n* = 4 PIs for each group.
(406 KB TIF)

Figure S4 Confirmation of Lesion of MB To confirm the hydroxyurea-induced ablation of MB, we expressed UAS-*GFP* ^S65T^ in MB (and a few other regions) using 201Y (201Y/UAS-*GFP*) PGAL4 drivers, or in projection neurons from ALs to MB using GH146 (GH146/UAS-*GFP*) PGAL4 drivers. Ten flies were sampled for the 201Y/UAS-*GFP* genotype with or without hydroxyurea treatment, and five flies were sampled for GH146/UAS-*GFP* with or without the treatment. In all cases, MB was ablated after hydroxyurea treatment, as indicated by the absence of UAS-*GFP* signal in MB calyces, consistent with previous reports [20,56].
(A) Without hydroxyurea treatment (−HU), UAS-*GFP* was expressed strongly in MB and a few scattered big neurons, and weakly in ALs with 201Y.
(B) Without hydroxyurea treatment (−HU), UAS-*GFP* was targeted to ALs and the projection neurons with GH146. Some weak GFP signal was also present in MB.
(C) The 201Y-driven GFP signal was reduced in MB specifically by hydroxyurea treatment (+HU), but still was present in the scattered neurons and ALs, suggesting the specific ablation of MB. The residual GFP signal in MB might represent the embryonic Kenyon cell fibers, unaffected by the treatment [57].
(D) The GH146-driven GFP signal was completely removed in MB by hydroxyurea (+HU). The hydroxyurea treatment also reduced GFP expression in ALs and the projection neurons, resulting from ablation of one lateral neuroblast [57,58].
(3.4 MB TIF)

Figure S5 Knockdown of the G_αq_ Protein There was a 50-bp overlap between the sequences used for creating UAS-ds*G_αq_* [36] and UAS-*dG_q_* ^1F1^ [37] transgenes. Therefore, Western blot analyses were done to confirm the RNAi-mediated disruption of G_αq_ protein with these two RNAi transgenes. Wild-type, UAS-ds*G_αq_*, or UAS-*dG_q_* ^1F1^ males were crossed with the Elav-PGAL4 virgins. Adult heads from their progenies were used for Western blot analyses. The quantification of six repetitions is shown in lower panel. G_αq_ expression was greatly disrupted in flies carrying both Elav-PGAL4 and UAS-ds*G_αq_* (dsG_αq_/Elav) or UAS-*dG_q_* ^1F1^ (dG_q_^1F1^/Elav) as compared with that in control flies carrying only Elav-PGAL4 driver.
(4.1 MB TIF)

Protocol S1 Detailed Description of Saturation Discrimination Assay in *Drosophila* and Supporting References (76 KB DOC)

Table S1 Olfactory Acuity to MCH and BA after Chemical Lesion of MB, Silencing of MB with shi^ts^, or Disruption of G_αq_ or G_αs_ Signaling in MB Chemical ablation of MB with hydroxyurea treatment (+HU), silencing of MB, or disruptions of G_αq_ or G_αs_ do not affect olfactory acuity. No significant differences were detected between MB-ablated (+HU) and control (−HU) flies (*p* ≥ 0.29), between +/shi^ts^ control flies and those with synaptic transmission from MB blocked (*p ≥* 0.21), between ds*G_αq_*/+ control flies and those with ds*G_αq_* expressed in MB (*p ≥* 0.39), or among genotypes overexpressing *G_αs_* ^+^ or G_αs_* (*p ≥* 0.23).
(60 KB DOC)

Table S2 Chemical Odors and Their Concentrations Used in the Saturation Discrimination Assay (51 KB DOC)

## Abbreviations

AL: antennal lobe
BA: benzaldehyde
*G*_α*s*_*: UAS-*G*_α*s*_^Q215L^
MB: mushroom body
MCH: 4-methyl-cyclohexanol
PI: performance index
RNAi: RNA interference
shi^ts^: UAS-*shi^ts1^*

## References

[pbio-0050264-b001] Wilson DA, Stevenson RJ (2003). The fundamental role of memory in olfactory perception. Trends Neurosci.

[pbio-0050264-b002] Ache BW, Young JM (2005). Olfaction: Diverse species, conserved principles. Neuron.

[pbio-0050264-b003] Shepherd GM (2005). Outline of a theory of olfactory processing and its relevance to humans. Chem Senses.

[pbio-0050264-b004] Linster C, Johnson BA, Morse A, Yue E, Leon M (2002). Spontaneous versus reinforced olfactory discriminations. J Neurosci.

[pbio-0050264-b005] Shimshek DR, Bus T, Kim J, Mihaljevic A, Mack V (2005). Enhanced odor discrimination and impaired olfactory memory by spatially controlled switch of AMPA receptors. PLoS Biol.

[pbio-0050264-b006] L'Etoile ND, Bargmann CI (2000). Olfaction and odor discrimination are mediated by the C. elegans guanylyl cyclase *ODR-1*. Neuron.

[pbio-0050264-b007] Kelliher KR, Ziesmann J, Munger SD, Reed RR, Zufall F (2003). Importance of the CNGA4 channel gene for odor discrimination and adaptation in behaving mice. Proc Natl Acad Sci U S A.

[pbio-0050264-b008] Kadohisa M, Wilson DA (2006). Olfactory cortical adaptation facilitates detection of odors against background. J Neurophysiol.

[pbio-0050264-b009] Devaud JM, Acebes A, Ferrus A (2001). Odor exposure causes central adaptation and morphological changes in selected olfactory glomeruli in *Drosophila*. J Neurosci.

[pbio-0050264-b010] Hildebrand JG, Shepherd GM (1997). Mechanisms of olfactory discrimination: Converging evidence for common principles across phyla. Annu Rev Neurosci.

[pbio-0050264-b011] Ronnett GV, Moon C (2002). G proteins and olfactory signal transduction. Annu Rev Physiol.

[pbio-0050264-b012] Mayford M, Kandel ER (1999). Genetic approaches to memory storage. Trends Genet.

[pbio-0050264-b013] Dubnau J, Tully T (1998). Gene discovery in *Drosophila*: New insights for learning and memory. Annu Rev Neurosci.

[pbio-0050264-b014] Gomez-Diaz C, Martin F, Alcorta E (2004). The cAMP transduction cascade mediates olfactory reception in Drosophila melanogaster. Behav Genet.

[pbio-0050264-b015] Gottfried JA, Winston JS, Dolan RJ (2006). Dissociable codes of odor quality and odorant structure in human piriform cortex. Neuron.

[pbio-0050264-b016] Savic I, Gulyas B, Larsson M, Roland P (2000). Olfactory functions are mediated by parallel and hierarchical processing. Neuron.

[pbio-0050264-b017] Barkai E, Hasselmo MH (1997). Acetylcholine and associative memory in the piriform cortex. Mol Neurobiol.

[pbio-0050264-b018] Quinlan EM, Lebel D, Brosh I, Barkai E (2004). A molecular mechanism for stabilization of learning-induced synaptic modifications. Neuron.

[pbio-0050264-b019] Connolly JB, Roberts IJ, Armstrong JD, Kaiser K, Forte M (1996). Associative learning disrupted by impaired Gs signaling in *Drosophila* mushroom bodies. Science.

[pbio-0050264-b020] de Belle JS, Heisenberg M (1994). Associative odor learning in *Drosophila* abolished by chemical ablation of mushroom bodies. Science.

[pbio-0050264-b021] McGuire SE, Le PT, Davis RL (2001). The role of *Drosophila* mushroom body signaling in olfactory memory. Science.

[pbio-0050264-b022] Dubnau J, Grady L, Kitamoto T, Tully T (2001). Disruption of neurotransmission in *Drosophila* mushroom body blocks retrieval but not acquisition of memory. Nature.

[pbio-0050264-b023] Wang Y, Guo HF, Pologruto TA, Hannan F, Hakker I (2004). Stereotyped odor-evoked activity in the mushroom body of *Drosophila* revealed by green fluorescent protein-based Ca^2+^ imaging. J Neurosci.

[pbio-0050264-b024] Wang Y, Wright NJ, Guo H, Xie Z, Svoboda K (2001). Genetic manipulation of the odor-evoked distributed neural activity in the *Drosophila* mushroom body. Neuron.

[pbio-0050264-b025] Zou Z, Buck LB (2006). Combinatorial effects of odorant mixes in olfactory cortex. Science.

[pbio-0050264-b026] Zou Z, Li F, Buck LB (2005). Odor maps in the olfactory cortex. Proc Natl Acad Sci U S A.

[pbio-0050264-b027] Illig KR, Haberly LB (2003). Odor-evoked activity is spatially distributed in piriform cortex. J Comp Neurol.

[pbio-0050264-b028] Stopfer M, Jayaraman V, Laurent G (2003). Intensity versus identity coding in an olfactory system. Neuron.

[pbio-0050264-b029] Carlson JR (1996). Olfaction in *Drosophila:* From odor to behavior. Trends Genet.

[pbio-0050264-b030] Tully T, Quinn WG (1985). Classical conditioning and retention in normal and mutant Drosophila melanogaster. J Comp Physiol [A].

[pbio-0050264-b031] Boynton S, Tully T (1992). *latheo,* a new gene involved in associative learning and memory in *Drosophila melanogaster,* identified from P element mutagenesis. Genetics.

[pbio-0050264-b032] Dura JM, Preat T, Tully T (1993). Identification of *linotte,* a new gene affecting learning and memory in Drosophila melanogaster. J Neurogenet.

[pbio-0050264-b033] Borst A (1983). Computation of olfactory signals in Drosophila melanogaster. J Comp Physiol [A].

[pbio-0050264-b034] Dudai Y (1977). Properties of learning and memory in Drosophila melanogaster. J Comp Physiol [A].

[pbio-0050264-b035] Kitamoto T (2001). Conditional modification of behavior in *Drosophila* by targeted expression of a temperature-sensitive shibire allele in defined neurons. J Neurobiol.

[pbio-0050264-b036] Kalidas S, Smith DP (2002). Novel genomic cDNA hybrids produce effective RNA interference in adult *Drosophila*. Neuron.

[pbio-0050264-b037] Banerjee S, Joshi R, Venkiteswaran G, Agrawal N, Srikanth S (2006). Compensation of inositol 1,4,5-trisphosphate receptor function by altering sarco-endoplasmic reticulum calcium ATPase activity in the *Drosophila* flight circuit. J Neurosci.

[pbio-0050264-b038] Zars T, Fischer M, Schulz R, Heisenberg M (2000). Localization of a short-term memory in *Drosophila*. Science.

[pbio-0050264-b039] Heimbeck G, Bugnon V, Gendre N, Keller A, Stocker RF (2001). A central neural circuit for experience-independent olfactory and courtship behavior in Drosophila melanogaster. Proc Natl Acad Sci U S A.

[pbio-0050264-b040] Stocker RF, Heimbeck G, Gendre N, de Belle JS (1997). Neuroblast ablation in *Drosophila* P[GAL4] lines reveals origins of olfactory interneurons. J Neurobiol.

[pbio-0050264-b041] Kulkarni MM, Booker M, Silver SJ, Friedman A, Hong P (2006). Evidence of off-target effects associated with long dsRNAs in Drosophila melanogaster cell-based assays. Nat Methods.

[pbio-0050264-b042] Ma Y, Creanga A, Lum L, Beachy PA (2006). Prevalence of off-target effects in *Drosophila* RNA interference screens. Nature.

[pbio-0050264-b043] Chen CN, Denome S, Davis RL (1986). Molecular analysis of cDNA clones and the corresponding genomic coding sequences of the Drosophila dunce
^+^ gene, the structural gene for cAMP phosphodiesterase. Proc Natl Acad Sci U S A.

[pbio-0050264-b044] Levin LR, Han PL, Hwang PM, Feinstein PG, Davis RL (1992). The *Drosophila* learning and memory gene rutabaga encodes a Ca^2+^/Calmodulin-responsive adenylyl cyclase. Cell.

[pbio-0050264-b045] Margulies C, Tully T, Dubnau J (2005). Deconstructing memory in *Drosophila*. Curr Biol.

[pbio-0050264-b046] Bargmann CI, Hartwieg E, Horvitz HR (1993). Odorant-selective genes and neurons mediate olfaction in C. elegans. Cell.

[pbio-0050264-b047] Lin HH, Lai JS, Chin AL, Chen YC, Chiang AS (2007). A map of olfactory representation in the *Drosophila* mushroom body. Cell.

[pbio-0050264-b048] Bargmann CI (2006). Comparative chemosensation from receptors to ecology. Nature.

[pbio-0050264-b049] Wang JW, Wong AM, Flores J, Vosshall LB, Axel R (2003). Two-photon calcium imaging reveals an odor-evoked map of activity in the fly brain. Cell.

[pbio-0050264-b050] Wang Y, Chiang AS, Xia S, Kitamoto T, Tully T (2003). Blockade of neurotransmission in *Drosophila* mushroom bodies impairs odor attraction, but not repulsion. Curr Biol.

[pbio-0050264-b051] Livingstone M, Hubel D (1988). Segregation of form, color, movement, and depth: Anatomy, physiology, and perception. Science.

[pbio-0050264-b052] Zeki S, Shipp S (1988). The functional logic of cortical connections. Nature.

[pbio-0050264-b053] Schreiner CE, Read HL, Sutter ML (2000). Modular organization of frequency integration in primary auditory cortex. Annu Rev Neurosci.

[pbio-0050264-b054] Tully T, Preat T, Boynton SC, Del Vecchio M (1994). Genetic dissection of consolidated memory in *Drosophila*. Cell.

[pbio-0050264-b055] Chiang AS, Liu YC, Chiu SL, Hu SH, Huang CY (2001). Three-dimensional mapping of brain neuropils in the cockroach, Diploptera punctata. J Comp Neurol.

[pbio-0050264-b056] McBride SM, Giuliani G, Choi C, Krause P, Correale D (1999). Mushroom body ablation impairs short-term memory and long-term memory of courtship conditioning in Drosophila melanogaster. Neuron.

[pbio-0050264-b057] Ito K, Hotta Y (1992). Proliferation pattern of postembryonic neuroblasts in the brain of Drosophila melanogaster. Dev Biol.

[pbio-0050264-b058] Truman JW, Bate M (1988). Spatial and temporal patterns of neurogenesis in the central nervous system of Drosophila melanogaster. Dev Biol.

