# Supplementary material for: Segregation of Odor Identity and Intensity during Odor Discrimination in Drosophila Mushroom Body
Source: PLoS Biol. 2007 Oct 2;5(10):e264. doi: 10.1371/journal.pbio.0050264 (PMC1994992; doi:10.1371/journal.pbio.0050264)
Supplement: Supplementary file 1 [file pbio.0050264.sd001.doc]

##### Supporting Information for

**Segregation of Odor Identity and Intensity during Odor Discrimination in *Drosophila* Mushroom Body**

Shouzhen Xia and Tim Tully*

To whom correspondence should be addressed. E-mail: [tully@cshl.edu](mailto:tully@cshl.edu)

**Supporting Information contains:**

Protocol S1, including a detailed description of saturation discrimination assay in *Drosophila* and supporting references

Table S1 to S2

Figure S1 to S5

**Protocol S1**

**Saturation discrimination assay**

Drosophila has long been used as a model system to study olfaction. Odors elicit a variety ***of behavioral responses via a relatively simple, but sensitive olfactory system readily accessible to genetics [1]. A number of behavioral assays have been reported for screening single-gene mutations defective in olfactory function. Most paradigms allow measuring a fly’s ability to sense individual odors, but none is appropriate for measuring olfactory discrimination between two odors. Probably the most popular paradigm for measuring olfactory perception of single odors in a T-maze is the olfactory acuity assay,*** in which naïve flies are allowed to choose between an aversive odor and air [2]. Flies respond very fast and usually make their choice within 2 minutes. They respond to a large number of odors including various alcohols, ketones, esters, aromatic compounds and amines over a wide range of concentrations (unpublished data; [2-4]). Interestingly, most of the tested odors are repulsive to flies at high but attractive at low concentrations (unpublished data; [4]).

Both olfactory perception of single odors and discrimination between different odors may be required for discriminative olfactory learning, and arguably depend on the integrity of normal memory [5]. During discriminative olfactory conditioning in *Drosophila*, flies are exposed sequentially to two different CS (conditioned stimulus) odors, with the first odor (CS+) paired with electric shock (US) [2]. Because the CS+ is much more salient (concentrated) than any potential background odors in the air current, minimizing the need for any odor discrimination during training, olfactory discrimination may not necessarily be involved with associative learning where perception of individual odors is sufficient. Conventionally, people use odor avoidance assay to check whether naïve flies can perceive single odors as compared with air. Subsequent olfactory perception measured as odor avoidance is increased for CS+ odor, but decreased for CS- (T.T., unpublished data). During testing, flies are given a choice in the T-maze between the CS+ and CS- odors presented simultaneously without reinforcement. In this context, flies need to discriminate between the two odors and then move away from the CS+ (if associative learning/memory has occurred). So, when screening for mutants defective in associative learning, defects in “discriminability” must be ruled out as a more trivial explanation for their performance deficits. Until very recently, however, this issue has not been addressed (current study; S.X. and T.T., in preparation).

To evaluate whether naïve flies can discriminate odor identity independent of odor intensity (concentration), we have developed an assay in which an intensity-saturated odor was delivered in both T-maze arms, and a second odor was delivered in only one T-maze arm. Saturating odor concentrations were determined behaviorally (Figure S2A). Naïve flies were allowed to choose in the T-maze between 1x [MCH] versus 2x [MCH]. Initially, flies would avoid the T-maze arm with the higher concentration, yielding a positive PI. As the concentration of MCH was increased, however, a threshold was reached, above which flies were unable to recognize the 1x versus 2x intensity difference, yielding a random distribution between the T-maze arms and a PI of zero. This represented the saturating concentration of MCH, MCH^S^ (15%). Next, flies were given a choice between MCH^S^ and varying concentrations of BA, again until the performance index was zero (Figure S2B). This determined the behaviorally equivalent concentration of BA, BA^E^. Finally, flies were given a choice between MCH^S^ + BA^E^ in one T-maze arm and 1x MCH^S^ in the other (Figure S2C, black bar marked with “*”). The resulting non-zero performance index indicates that flies were able to discriminate BA from MCH, independent of odor intensity. A similar effect was seen when flies were given a choice between MCH^S^ + BA^E^ and 2x MCH^S^ (Figure S2C, grey bar), confirming that discrimination of odor identity was occurring at saturating concentrations of odor intensity.

In the “2 x MCH^S^ versus MCH^S^” test, the actual vapor concentration of 2 x MCH^S^ was higher than that of MCH^S^ but far less than 2 times of the vapor concentration of MCH^S^ (Figure S2C, lower panel). This suggests that evaporation of MCH^S^ was not saturated, and an intensity difference was indeed present in the test where flies failed to detect it. The vapor concentration of BA^E^ was pretty close to that of MCH^S^. This is surprising, because the concentration (15%) of MCH^S^ is about 10 times of that (1.5%) of BA^E^. The total vapor concentration of MCH^S^ + BA^E^ (63 ± 1.2 PPM) is close to the additive total of the vapor concentrations of MCH^S^ (33.9 ± 1.1 PPM) and BA^E^ (35.5 ± 1.4 PPM). Therefore, the effective concentration of MCH^S^ was not changed in the mixture of MCH^S^ and BA^E^. The vapor concentration difference between MCH^S^ + BA^E^ and MCH^S^ was much bigger than that between MCH^S^ + BA^E^ and 2xMCH^S^, but flies produced similar behavioral scores in both the “MCH^S^ + BA^E^ versus MCH^S^” and “MCH^S^ + BA^E^ versus 2xMCH^S^” tests. This suggests that the intensity difference present in the two tests contributes little to the choice behavior of flies and therefore discrimination of odor identity occurs independent of odor intensity.

Spontaneous odor identity discrimination was concentration-dependent (Figure S2D). Naïve flies were less able to discriminate lower concentrations of BA in the context of the saturating concentration of MCH. The resulting performance indices were dramatically lower than those produced in the olfactory acuity assay at corresponding concentrations (Table S1). Under these conditions, optimal choice behavior in the T-maze occurred within 30s (Figure S2E), after which performance indices dropped. Presumably, olfactory responses adapted somewhat at these higher odor concentrations. Consistent with this notion, the optimal odor concentrations for the discrimination of odor identity in conditioned flies (see below) were 10^-3^ for MCH and 0.4 x 10^-3^ for BA, which yielded a low performance index in this saturation assay (Figure S2D).

Naïve flies were able to discriminate chemically different but not similar odors in this spontaneous odor identity assay. When asked to discriminate (R)-(-)-carvone from (S)-(+)-carvone as the background, (R)-(-)-octanol from (S)-(+)-octanol as the background, pentanol or 2-hexanol from hexanol as the background, flies produced performance scores close to zero (data not shown). These observations are consistent with previous reports for other animals [6]. When tested for their ability to discriminate 3-octonal, benzaldehyde (BA), ethyl acetate, diacetyl, triethylamine and thiazole from MCH^S^, as well as MCH, 3-octonal, ethyl acetate, diacetyl, triethylamine and thiazole from BA^S^ (see Table S2 for concentrations of all odors), flies produced variable scores in the range of -90 to 80, depending on the particular combinations of odors used (Figure S3A). An interesting exception was the low score produce by discrimination of 3-octonal from MCH, which are distinct chemical classes and which are used routinely in conditioned odor identity assay.

Surprisingly, the performance indices were negative for some odor pairs, indicating that flies avoided the saturating background odors in favor of odor mixtures. It remains unclear how odor mixtures are perceived, especially at the MB level. Mixtures of two or more odors frequently give rise to novel perceptual qualities that are not present in any of the components individually (e.g., [7]). In mammals, this may be due to novel cortical representations in olfactory cortex induced by mixtures [8]. Flies, however, may use the repulsiveness of odors to guide their choice behavior. Among all these odors, saturated thiazole was the strongest repulsive odor, followed by ethyl acetate, BA, diacetyl, MCH, 3-octonal and triethylamine. Saturated ethyl acetate, BA and diacetyl were similar to each other in repulsiveness, and triethylamine appeared neutral even as a pure odor (data not shown). Adding a second “foreground” odor into a more repulsive background seemed to make it less repulsive (but still very repulsive, data not shown), and flies chose the mixtures over the background alone. Reciprocally, when the background odors were less repulsive, flies chose the background alone over mixtures. With BA as the background, flies chose mixtures with MCH, 3-octonal, and triethylamine over BA alone. With MCH as the background, flies preferred mixtures when with 3-octonal or triethylamine were discriminated. This suggests that mixing a more repulsive background odor with a less repulsive one makes it less repulsive, and flies always avoid the more repulsive odors or mixtures in the saturation assay.

Reciprocal experiments further supported this notion (Figure S3B). When a more repulsive odor such as thiazole was the saturating background, flies would prefer mixtures of thiazole and MCH or BA versus thiazole alone. When a less repulsive odor such as 3-octonal is the saturating background, the animals would prefer 3-octonal alone rather than mixtures of 3-octonal and MCH or BA. Furthermore, when allowed to discriminate MCH from ethyl acetate and diacetyl, the animals chose the mixtures over the background alone. Finally, when two odors were equivalent or similar in repulsiveness such as BA and diacetyl, the animals appeared to prefer the background alone over the mixture (Figure S3A-B). Therefore, naïve flies are able to discriminate odors from a chemically different odor as the saturated background, and most likely avoid the relatively more repulsive arm which may be the background alone (by itself less repulsive than the foreground odors to be discriminated) or the mixtures (more repulsive).

The saturation assay is a measure of spontaneous odor identity discrimination in naïve flies. First, the assay involves a saturated background odor in both T-maze arms and a second foreground odor, which can be saturated or unsaturated, present only in one T-maze arm. Flies need to recognize the presence of the foreground odor and thus discriminate it from the background to perform properly in the assay. As compared with the saturated background odor, the foreground odor may be different in two possible ways. The odor may activate additional receptors, leading to activation of additional subsets of glomeruli (and eventually MB neurons). Alternatively, an odor may activate additional signaling pathways, leading to activation of an additional subset of MB neurons. The low performance for saturation tests between chemically similar odors but high scores between chemically different odors are consistent with idea that odor identity discrimination exists. Secondly, the assay is very different from the olfactory acuity assay where perception of single odors (vs. air) is evaluated. Odor identity discrimination requires normal function of MBs, while olfactory acuity not (Figure 1, Table S1). More specifically, it requires *G_αq_* signaling in MB (Figure 2), while perception *G_αq_* signaling upstream of MB [9]. Interestingly, both processes seem to recruit overlapping sets of genes. Three different types of olfactory mutants have been identified: one group with abnormal performance in both tests, a second group with defects only in olfactory acuity, and a third group with defective spontaneous odor identity discrimination (S.X. and T.T., unpublished data). Finally, the spontaneous odor identity discrimination assay is different from the conditioning odor identity discrimination assay where trained flies are allowed to discriminate two odors which naïve animals do not readily discriminate. Both assays require the normal functional of MB, but recruit different G-protein signaling pathways within MBs. *G_αq_* subserves spontaneous odor identity discrimination, while *G_as_* subserves conditioned odor identity discrimination (Figures 2, 4). Here too, evidence suggests a “genetic dissection” of the two assays. Most of the known learning and memory mutants display normal spontaneous odor identity discrimination (S.X. and T.T., in preparation).

**Supporting References**

1.	Carlson JR (1996) Olfaction in Drosophila: from odor to behavior. Trends Genet 12: 175-180.

2.	Tully T, Quinn WG (1985) Classical conditioning and retention in normal and mutant Drosophila melanogaster. J Comp Physiol [A] 157: 263-277.

3.	Ayer RK Jr, Carlson J (1992) Olfactory physiology in the Drosophila antenna and maxillary palp: acj6 distinguishes two classes of odorant pathways. J Neurobiol 23: 965-982.

4.	Wang Y, Wright NJ, Guo H, Xie Z, Svoboda K, Malinow R, Smith DP, Zhong Y (2001) Genetic manipulation of the odor-evoked distributed neural activity in the Drosophila mushroom body. Neuron 29: 267-276.

5.	Wilson DA, Stevenson RJ (2003) The fundamental role of memory in olfactory perception. Trends Neurosci 26: 243-247.

6.	Linster C, Johnson BA, Morse A, Yue E, Leon M (2002) Spontaneous versus reinforced olfactory discriminations. J Neurosci 22: 6842-6845.

7.	Firestein S (2004) A code in the nose. Sci STKE 2004: pe15.

8.	Zou Z, Buck LB (2006) Combinatorial effects of odorant mixes in olfactory cortex. Science 311: 1477-1481.

9.	Kalidas S, Smith DP (2002) Novel genomic cDNA hybrids produce effective RNA interference in adult Drosophila. Neuron 33: 177-184.
