# Supplementary material for: Segregation of Odor Identity and Intensity during Odor Discrimination in Drosophila Mushroom Body
Source: PLoS Biol. 2007 Oct 2;5(10):e264. doi: 10.1371/journal.pbio.0050264 (PMC1994992; doi:10.1371/journal.pbio.0050264)
Supplement: Supplementary file 2 [file pbio.0050264.st001.doc]

Table S1

| Flies | Concentration of MCH (v/v) | | | | Concentration of BA (v/v) | | | |
| --- | --- | --- | --- | --- | --- | --- | --- | --- |
|  | 0.0001 | 0.001 | 0.01 | 0.15 | 0.00004 | 0.0004 | 0.015 | 0.1 |
| − HU | 44 ± 5 | 73 ± 3 | 94 ± 1 | 96 ± 1 | 39 ± 6 | 77 ± 2 | 95 ± 1 | 98 ± 1 |
| + HU | 41 ± 4 | 68 ± 4 | 92 ± 2 | 96 ± 1 | 33 ± 6 | 81 ± 2 | 93 ± 2 | 98 ± 1 |
| +/shi^ts^ | 34 ± 6 | 66 ± 5 | 88 ± 3 | 95 ± 2 | 36 ± 6 | 68 ± 3 | 93 ± 1 | 96 ± 1 |
| C309/shi^ts^ | 29 ± 6 | 58 ± 6 | 89 ± 2 | 95 ± 1 | 30 ± 6 | 62 ± 3 | 91 ± 3 | 95 ± 2 |
| 247/shi^ts^ | 38 ± 6 | 63 ± 4 | 90 ± 3 | 93 ± 1 | 32 ± 7 | 69 ± 4 | 90 ± 2 | 95 ± 1 |
| 201Y/shi^ts^ | 27 ± 6 | 60 ± 6 | 88 ± 2 | 94 ± 2 | 35 ± 8 | 60 ± 4 | 89 ± 3 | 97 ± 1 |
| dsG_αq_/+ | 40 ± 5 | 72 ± 3 | 86 ± 2 | 96 ± 1 | 30 ± 4 | 72 ± 3 | 90 ± 2 | 97 ± 1 |
| dsG_αq_/C309 | 36 ± 5 | 65 ± 4 | 87 ± 2 | 95 ± 2 | 24 ± 5 | 67 ± 4 | 90 ± 2 | 98 ± 1 |
| dsG_αq_/247 | 32 ± 4 | 67 ± 4 | 88 ± 2 | 97 ± 1 | 25 ± 5 | 64 ± 4 | 87 ± 2 | 97 ± 1 |
| dsG_αq_/201Y | 30 ± 7 | 63 ± 6 | 86 ± 2 | 97 ± 1 | 34 ± 5 | 70 ± 3 | 93 ± 2 | 98 ± 1 |
| G_αs_^+^/C309 | 42 ± 4 | 76 ± 3 | 87 ± 2 | 97 ± 1 | 33 ± 6 | 70 ± 4 | 94 ± 1 | 97 ± 1 |
| G_αs_^*^/C309 | 36 ± 4 | 79 ± 2 | 84 ± 3 | 96 ± 1 | 28 ± 4 | 63 ± 3 | 94 ± 1 | 97 ± 1 |
| G_αs_^+^/247 | 37 ± 5 | 73 ± 4 | 85 ± 2 | 97 ± 1 | 39 ± 5 | 71 ± 3 | 94 ± 1 | 98 ± 1 |
| G_αs_^*^/247 | 31 ± 1 | 68 ± 4 | 82 ± 3 | 97 ± 1 | 33 ± 8 | 74 ± 4 | 92 ± 2 | 97 ± 1 |
| G_αs_^+^/201Y | 39 ± 4 | 66 ± 5 | 86 ± 2 | 96 ± 1 | 37 ± 6 | 74 ± 4 | 93 ± 1 | 98 ± 1 |
| G_αs_^*^/201Y | 36 ± 5 | 72 ± 4 | 84 ± 2 | 97 ± 1 | 27 ± 5 | 69 ± 3 | 92 ± 1 | 98 ± 1 |
