# Supplementary material for: Segregation of Odor Identity and Intensity during Odor Discrimination in Drosophila Mushroom Body
Source: PLoS Biol. 2007 Oct 2;5(10):e264. doi: 10.1371/journal.pbio.0050264 (PMC1994992; doi:10.1371/journal.pbio.0050264)
Supplement: Supplementary file 3 [file pbio.0050264.st002.doc]

Table S2

| B/A | | A: saturated concentration of the background odor A (%) | | | | | |
| --- | --- | --- | --- | --- | --- | --- | --- |
|  |  | MCH | 3-octonal | BA | diacetyl | ethyl acetyl | thiazole |
| B: equivalent concentration of discriminative odor B to the saturated background A (%) | MCH | 15/15 | 8/40 | 15/10 | 20/15 | 20/15 | 20/10 |
|  | 3-octonal | 20/15 | 40/40 | 40/10 | ⎯ | ⎯ | ⎯ |
|  | BA | 1.5/15 | 0.6/40 | 10/10 | 10/15 | 10/15 | 10/10 |
|  | diacetyl | 2.0/15 | ⎯ | 10/10 | 15/15 | ⎯ | ⎯ |
|  | ethyl acetyl | 2.0/15 | ⎯ | 15/10 | ⎯ | 15/15 | ⎯ |
|  | triethylamine | 40/15 | ⎯ | 40/10 | ⎯ | ⎯ | ⎯ |
|  | thiazole | 1.0/15 | ⎯ | 5/10 | ⎯ | ⎯ | 10/10 |

**Supporting References**

1.	Carlson JR (1996) Olfaction in Drosophila: from odor to behavior. Trends Genet 12: 175-180.

2.	Tully T, Quinn, WG (1985) Classical conditioning and retention in normal and mutant Drosophila melanogaster. J Comp Physiol [A] 157: 263-277.

3.	Ayer RK Jr, Carlson J (1992) Olfactory physiology in the Drosophila antenna and maxillary palp: acj6 distinguishes two classes of odorant pathways. J Neurobiol 23: 965-982.

4.	Wang Y, Wright NJ, Guo HF, Xie Z, Svoboda K, et al. (2001) Genetic manipulation of the odor-evoked distributed neural activity in the Drosophila mushroom body. Neuron 29: 267-276.

5.	Wilson DA, Stevenson RJ (2003) The fundamental role of memory in olfactory perception. Trends Neurosci 26: 243-247.

6.	Linster C, Johnson BA, Morse A, Yue E, Leon M (2002) Spontaneous versus reinforced olfactory discriminations. J Neurosci 22: 6842-6845.

7.	Firestein S (2004) A code in the nose. Sci STKE 2004: pe15.

8.	Zou Z, Buck LB (2006) Combinatorial effects of odorant mixes in olfactory cortex. Science 311: 1477-1481.

9.	Kalidas S, Smith DP (2002) Novel genomic cDNA hybrids produce effective RNA interference in adult Drosophila. Neuron 33: 177-184.

10.	de Belle JS, Heisenberg M (1994) Associative odor learning in Drosophila abolished by chemical ablation of mushroom bodies. Science 263: 692-695.

11.	McBride SM, Giuliani G, Choi C, Krause P, Correale D, et al. (1999) Mushroom body ablation impairs short-term memory and long-term memory of courtship conditioning in Drosophila melanogaster. Neuron 24: 967-977.

12.	Ito K, Hotta Y (1992) Proliferation pattern of postembryonic neuroblasts in the brain of Drosophila melanogaster. Dev Biol 149: 134-148.

13.	Truman JW, Bate M (1988) Spatial and temporal patterns of neurogenesis in the central nervous system of Drosophila melanogaster. Dev Biol 125: 145-157.

14.	Banerjee S, Joshi R, Venkiteswaran G, Agrawal N, Srikanth S, et al. (2006) Compensation of inositol 1,4,5-trisphosphate receptor function by altering sarco-endoplasmic reticulum calcium ATPase activity in the Drosophila flight circuit. J Neurosci 26: 8278-8288.
